# Editorial: Novel small molecules in targeted cancer therapy

**DOI:** 10.3389/fphar.2023.1272523

**Published:** 2023-08-15

**Authors:** Rong Li, Xian-Li Ma, Chao Gou, William Ka Fai Tse

**Affiliations:** ^1^ Key Laboratory of Environmental Pollution and Integrative Omics, Education Department of Guangxi Zhuang Autonomous Region, Guilin Medical University, Guilin, China; ^2^ Department of Pharmacy, Guilin Medical University, Guilin, China; ^3^ Department of Clinical Pharmacy, Guigang City People’s Hospital, The Eighth Affiliated Hospital of Guangxi Medical University, Guigang, China; ^4^ Laboratory of Developmental Disorders and Toxicology, Center for Promotion of International Education and Research, Faculty of Agriculture, Kyushu University, Fukuoka, Japan

**Keywords:** small molecules, cancer, cell death, metastasis, molecular mechanism, pharmacology, targeted therapy

## Introduction

Over the last few decades, small molecule cancer drug discovery and development have focused on personalized medicine, emphasizing molecularly targeted drugs. Because of their small size (usually ≤500 Da), small molecules have been successfully used to target the extracellular, cell surface ligand-binding receptors and the intracellular proteins that play key roles in transducing downstream signalling for cancer cell survival, proliferation, and metastasis. Small molecule cancer drugs cover a wide range of inhibitors, including kinase inhibitors, proteasome inhibitors, matrix metalloproteinase inhibitors, etc. Research on molecularly targeted cancer drug discovery has resulted in some small molecule drugs being successfully introduced in the clinic for cancer treatment. Although many small molecules cancer drugs have been developed and are currently being utilized in the clinic, many challenges remain, such as a low response rate and drug resistance.

The current Research Topic would like to raise such issues for discussion; and how we could overcome the problems by understanding the mechanisms underlying the effect of small molecule cancer drugs. This Research Topic is under the Pharmacology of Anti-Cancer Drugs section of the journal Frontiers in Pharmacology, a sum of seven contributing articles (6 research articles, and 1 review article) have been included, and we would like to summarize their findings in this editorial article. The works can be divided into two categories, based on their experimental approaches.

### Biological experiments

In this Research Topic, three types of cancers have been studied by various compounds. Qu et al. aimed to study the known anti-tumor compound, calycosin, on thyroid cancer. Calycosin is one of the main components of astragalus, which could induce the apoptosis via PI3K/AKT/mTOR signaling pathway. However, its anti-cancer mechanism on papillary thyroid cancer has not been fully described. Authors underwent the microarray analysis on the B-CPAP cells and, confirmed that calycosin could influence the apoptotic genes and p70S6 Kinase via the Sestrin2/AMP-activated protein kinase/mammalian target of rapamycin complex (SESN2/AKT/mTOR) pathway.

A study on colorectal cancer by Otkur et al. aimed to find out if the orphan G_protein coupled receptor GPR35 antagonists could serve as a good candidate in inhibiting the colorectal cancer. They showed that the GPR35 could stimulate the YAP/TAZ activity in Hippo signaling pathway through the Rho-GTPase. Besides, they identified that the GPR35 antagonist CID-2745687 could suppress the hyperactivation of YAP/TAZ in colorectal cancer.

Non-small cell lung cancer is the major types of lung cancers that has a very low 5-year overall survival rate. Hu et al. here purposed the anti-tumor feature of N-linoleyltyrosine (NlTyr), an anandamide analog, in A549 cells. They found that NlTyr could active the endocannabinoid receptors via the PI3K and ERK pathways, which resulted in cell cycle arrest, inhibited migration, and induced apoptosis in the non-small cell lung cancer cells.

One review paper on the potential use of p21-activated kinase 4 (PAK4) inhibitors was published in this Research Topic. Li et al. summarized the current findings on the PAK4 mediated singaling pathways, and their roles in cancers. The article described the potential anti-tumor abilities of PAK4 inhibitors, and classified them into ATP competitive inhibitors, and allosteric inhibitors. They further investigated the preclinical and clinical studies of two selected inhibitors, PF-3758309 and KPT-9274, which was terminated and in phase I clinical trials, respectively. The article ended with the upcoming challenges, limitations, and prospective on PAK4 usage.

### Network pharmacology, database screening, and meta-analysis

Network pharmacology is a powerful tool to characterize the action mechanisms of undeveloped or complicated derivatives in drug discovery. Chen et al. applied the network pharmacology-based prediction and *in vitro* cell line models on the coumarin derivatives and demonstrated that the compound 6e could suppress migration, invasion, and induce mitochondria dependent apoptosis via the PI3K/AKT-mediated BCL-2 signaling pathway.

Taghvaei et al. performed the *in silico* database screening that targeting on the Sentrin-specific protease 1 (SENP1). SENP1 participates in cancer development by inhibit the apoptosis, and stimulate the cell proliferation and migration. Besides, it promotes the angiogenesis that shape it as a common target for cancer drugs. The group screened for 84,000 compounds and selected final 20 compounds after the molecular docking analysis. They then identified that resveratrol and ZINC33916875 could act as the SENP1 inhibitor after the essential dynamics and molecular dynamics analysis.

Cai et al. aimed to understand the clinical benefit of different regiments (double-drug or triple-drug) for left-sided RAS wild-type metastatic colorectal cancer patients. They performed a network meta-analysis by using the published data and real-world data, and concluded that a double-drug regimen plus cetuximab/panitumumab is more effective than double-drug- and triple-drug-based regimens with bevacizumab for left-sided RAS wild-type metastatic colorectal cancer. Besides, they claimed that the double-drug regimen plus cetuximab/panitumumab is safer than triple-drug regimen with bevacizumab via the safety analysis.

To summarize, the Research Topic has provided several new potential anti-cancer compounds by various approaches, such as performing meta-analysis on patient data to identify the clinical benefit of treatments; biological experiments in cell models to identify the molecular mechanism of the compounds; computational analysis on compounds’ structure and docking abilities. We hope that the Research Topic could provide some directions to researchers for searching the new anti-cancer drugs.

